# A Comparison of Biocompatibility of a Titanium Alloy Fabricated by Electron Beam Melting and Selective Laser Melting

**DOI:** 10.1371/journal.pone.0158513

**Published:** 2016-07-08

**Authors:** Hong Wang, Bingjing Zhao, Changkui Liu, Chao Wang, Xinying Tan, Min Hu

**Affiliations:** 1 Department of Stomatology, General Hospital of the People’s Libration Army, Beijing, China; 2 Department of Stomatology, the Second Affiliated Stomatological Hospital of Liaoning Medical University, Jinzhou, China; 3 Department of Stomatology, the 451th Hospital of the People’s Libration Army, Xi’an, China; 4 School of Medicine, Nankai University, Tianjin, China; 5 Department of Stomatology, the 304th Hospital of the People’s Libration Army, Beijing, China; University of Akron, UNITED STATES

## Abstract

Electron beam melting (EBM) and selective laser melting (SLM) are two advanced rapid prototyping manufacturing technologies capable of fabricating complex structures and geometric shapes from metallic materials using computer tomography (CT) and Computer-aided Design (CAD) data. Compared to traditional technologies used for metallic products, EBM and SLM alter the mechanical, physical and chemical properties, which are closely related to the biocompatibility of metallic products. In this study, we evaluate and compare the biocompatibility, including cytocompatibility, haemocompatibility, skin irritation and skin sensitivity of Ti6Al4V fabricated by EBM and SLM. The results were analysed using one-way ANOVA and Tukey’s multiple comparison test. Both the EBM and SLM Ti6Al4V exhibited good cytobiocompatibility. The haemolytic ratios of the SLM and EBM were 2.24% and 2.46%, respectively, which demonstrated good haemocompatibility. The EBM and SLM Ti6Al4V samples showed no dermal irritation when exposed to rabbits. In a delayed hypersensitivity test, no skin allergic reaction from the EBM or the SLM Ti6Al4V was observed in guinea pigs. Based on these results, Ti6Al4V fabricated by EBM and SLM were good cytobiocompatible, haemocompatible, non-irritant and non-sensitizing materials. Although the data for cell adhesion, proliferation, ALP activity and the haemolytic ratio was higher for the SLM group, there were no significant differences between the different manufacturing methods.

## Introduction

Rapid prototyping (RP) is a series of advanced manufacturing technologies and is being implemented in industrial and biomedical areas [1–5]. Electron beam melting (EBM) and selective laser melting (SLM) are two advanced types of RP and direct metal melting layer manufacturing technologies [6–9]. EBM and SLM enable the direct fabrication of complex structures and geometric shapes using computer-aid-design (CAD) without any tooling, which saves time and is highly effective. The fabrication processes for EBM and SLM are to selectively melt raw powder materials with either an electron beam or a focused laser based on the data in the part’s associated CAD file. Due to the different fabrication processes, the microstructure, mechanical and chemical properties of EBM and SLM metal products are different from those fabricated from wrought, cast or powder metallurgy materials [10–15]. Furthermore, different fabrication parameters, including power size, scan speed and building speed between the EBM and SLM systems result in different microstructures as well. Thijs et al and Sallica-Leva et al demonstrated that a SLM Ti6Al4V sample exhibited a very fine aciclar martensite grain structure [10–11]. Murr et al demonstrated that an EBM Ti6Al4V sample had a uniform, acicular α-phase microstructure (with β-phase), which was similar to a wrought product [12]. Koike et al described EBM Ti6Al4V that consisted of prominent acicular α-plates and SLM Ti6Al4V that consisted of a mixture of α-phase and α’ martensite [13]. In addition, the microstructure is related to the mechanical and chemical properties of metal. Rafi et al showed that the tensile strength and fatigue properties in SLM Ti6Al4V samples is higher than for EBM Ti6Al4V samples [14]. They attributed the difference in mechanical properties to the differences in the microstructures. Koike et al discussed how SLM Ti6Al4V exhibited better corrosion resistance than EBM Ti6Al4V. This result was from the acicular α-plates in the α-phase dominating in the EBM specimen to a greater extent than the α’ martensite in the SLM specimen [13]. Due to the high efficiency, lack of tooling required, complex geometric structures capable of being fabricated from CT or CAD data, EBM and SLM are two superior metal manufacturing methods for medical applications. Good biocompatibility is the basic requirement for any clinical application of a medical material. Metallic medical implants remain in long-term contact with bodily fluids and tissues, which may lead corrosion and the release of alloying elements into the body. The release of alloying elements causing adverse effects has been investigated in [16–18]. Accordingly, the biocompatibility of SLM and EBM Ti6Al4V must be investigated prior to clinical applications. Warnke et al and Kawase et al summarized that SLM Ti6Al4V products had good biocompatibility and were suitable for medical applications [19–20]. Studies from Peppo et al and Harbe et al demonstrated that an EBM titanium alloy supports cell attachment, growth and differentiation [21–22]. Nevertheless, there have been few investigations on the comparison of the biocompatibility between EBM and SLM products. In this study, we assessed and compared the in vitro and in vivo biocompatibility of Ti6Al4V fabricated by EBM and SLM. Commercial medical Ti6Al4V was employed as a control.

## Materials and Methods

### Animals

All the experiments were approved by the Institutional Animal Care and Use Committee of the General Hospital of the People's Liberation Army and were conducted following its guidelines.

A 2-year-old, healthy beagle (Beijing Marshall Biotechnology Co, China) with a weight of 10 kg was housed in a cage, placed in a temperature controlled room and received a standard diet as well as water ad libitum.

Four adult, healthy and female New Zealand white rabbits (Vital River Laboratory Animal Technology Co. Ltd, China) were housed individually in cages in a clean room exclusively constructed for rabbits and fed fresh rabbit food and water ad libitum.

Thirty adult, healthy albino guinea pigs (Vital River Laboratory Animal Technology Co. Ltd, China), female (nulliparous and not pregnant), with weights between 300–350 g were housed individually in cages with aseptic padding and given a standard pellet diet and water ad libitum. All the animals were sacrificed after testing with an overdose of pentobarbital sodium (3%) injection, except for the beagle, which was used in another study after bone marrow collection.

### Preparation of the Ti6AL4V specimens

The Ti6AL4V specimens were divided into three groups in this study based on fabrication technique:

Wrought: Ti6AL4V worked specimens (China),
EBM: Ti6AL4V specimens produced by EBM using an Arcam EBM A1 system and Arcam Ti6Al4V (Grade 5) powder with particle sizes from 45 to 100 μm (Arcam AB, Molndal, Sweden),
SLM: Ti6AL4V specimens produced by SLM using an EOS M280 system and Ti6Al4V powder with an average particle size of 20 μm (EOS M280, Germany). The Ti6AL4V specimens were washed with acetone, dehydrated alcohol and triple distilled water with an ultrasonic washing unit for 10 minutes. The process was performed twice. Sterilization of the Ti6AL4V specimens was conducted using high pressure sterilizer (YXQ-LS-100A, China) 121(°C) for 20 min.

### Microstructures of Ti6AL4V specimens

The EBM and SLM Ti6AL4V specimens were mounted, mechanically polished and etched in Kroll’s reagent. The microstructures were examined by an optical microscope (Zeiss Axiovert 200 MAT, Germany).

### Cytocompatibility examination

#### MSCs isolated and identification

General anaesthesia of the beagle was induced using 3% pentobarbital sodium (1 mL/kg) intravenously and 2% (w/v) Xylazine hydrochloride intramuscularly (7 mg/kg) to reduce pain. Bone marrow was harvested from the iliac crest with a sterile Rosenthal paediatric needle (16-gauge). Mesenchymal stem cells (MSCs) were isolated from the bone marrow and incubated in a 75 cm^2^ culture flask (Corning, Costar, NY, USA) with low-glucose Dulbecco’s Modified Eagle Medium (DMEM, Gibco, Invitrogen, Australia) supplemented with 100 U/mL penicillin (Gibco), 100 μg/mL streptomycin (Gibco) and 10% foetal bovine serum (FBS, Gibco) at 37°C in a humid atmosphere of 5% CO2 and 95% air. The cell surface markers for the MSCs showed that CD44(FITC) and CD90(APC) were positive. In contrast, CD34 (PE) and CD45 (FITC) (anti-canine, eBioscience, USA) were negative, as identified by flow cytometry (FACSCalibur, BD, USA). The osteogenic and adipogenic potentials were identified by positive staining of the MSCs in alizarin red staining and oil red O staining after osteogenic and adipogenic induction, respectively (Cyagen, Cyagen Biosciences Inc,USA) for 14 days. These results demonstrated that cells isolated from bone marrow possess multi-lineage differentiation (these data are not shown here). Passage 3 MSCs were applied to assess the response from the Ti6AL4V specimens.

#### Cell proliferation assessed by Cell Count

The Ti6AL4V specimens used in this test were quadrate, with an approximate diameter of 10 mm, a thickness of 2 mm, polished using 600 grit emery paper, washed and sterilized. Three aseptic specimens from each group were soaked in 12-well plates with DMEM without FBS overnight. A 500 μL passage 3 MSCs with a density of 10^6^/mL were seeded on the specimens and 500 μL DMEM with 10% FBS was added into each well. After being incubated for 1, 3 and 7 days, the cells on each specimen were gently rinsed with a phosphate saline buffer (PBS) twice and detached with 0.25% trypsin/EDTA (Gibco). Cell concentration was examined by cell count using a cytometer (Count Star, USA).

#### Cell vitality evaluated by CCK-8

Leaching liquor for the Ti6AL4V specimens in each group was prepared according to the ISO 10993–12:2002 standard, with 1.25 mL/cm^2^ DMEM at 37°C for 24 h [23]. MSCs (5×10^3^ cells/well) were seeded in 96-well plate (Corning, USA) and incubated for 24 h. Then, the old media was removed and 100 μL of leaching liquor of each group was added into a 96-well plate and incubated for 1, 2 and 3 days. The normal medium was as a control. At each time point, 10 μL per well CCK-8 solution reagent (Dojindo Laboratories, Kumamoto, Japan) was added into the 96-well plate, after being incubated at 37°C for 3 h. The cell viability was measured using ultraviolet spectrophotometer (GE Healthcare, Sweden) at 490 nm.

#### Cell morphology with SEM

The Ti6AL4V specimens used in this test were quadrate, with an approximate diameter of 5 mm, a thickness of 2 mm, polished using 600 grit emery paper, washed and sterilized. Samples from each group were soaked in 24-well plates with DMEM. A volume of 500 μL MSCs with a density of 4×10^5^ cells were seeded on the specimens per well. A volume of 500 μL DMEM with 10% FBS was added into each well and incubated for 1, 3 and 7 days. At each time point, the cell seeding specimens were washed gently three times with PBS and fixed with 2% glutaraldehyde (pH 7.2) for 3 h at 4°C, and then washed three times with PBS. The samples were dehydrated using increasing concentrations of ethanol (40%, 70%, 90% and 100%), and then dried at the critical point drying, platinum sputtered and observed using scanning electron microscopy (TM3000, Japan).

#### ALP activity evaluation

Osteogenic leaching liquor was prepared using a 1.25 mL/cm^2^ osteogenic medium at 37°C for 24 h. MSCs were seeded on the 6-well plates with a density of 10^5^ cells per well and incubated for 1 day, instead of the osteogenic leaching liquor and incubated for 3, 7 and 14 days. The normal osteogenic medium was used as a control. Cells were harvested at each time point. Cell protein content concentrations were quantified with a BCA Protein Assay Kit (Sigma, USA). The ALP activity was measured with an ALP quantity kit (SK3051, Shanghai, China) to determine the standard ALP activity value (ALP activity/protein content concentration).

#### Osteogenic gene expression quantification analysis

Osteogenic differentiation ability was evaluated by analysing the osteogenic gene expression of the MSC cells using real-time PCR. Gene primer sequences (Table 1) were designed using the Primer Express 3.0 software (Applied Biosystems, Darmstadt, Germany). MSCs were seeded on 6-well plates with a density of 2×10^5^ per well and incubated with osteogenic leaching liquor for 1, 2 and 3 weeks, respectively. Afterwards, the cells were harvested and the total RNA was extracted using TriZol (Invitrogen, USA). The expression of the osteogenic differentiation related genes including Collage type one (COLI), RUNX2, ostecalcin (OCN) and osteopontin (OPN) was measured by real-time quantitative polymerase chain reaction (qPCR).

**Table 1:** Primer sequence for qRT-PCR.

Gene	Sequence
Actin-canis-F	5' GTGCGTGACATCAAGGAGAAGC 3'
Actin-canis-R	5' CTCAGGAAGGAAGGCTGGAAGA 3'
Runx2-canis-F	5' CGAAATGCCTCTGCTGTTATGA 3'
Runx2-canis-R	5' GGGGTCCATCCACTGTAACTTTA 3'
OPN-canis-F	5' TCCGATGAACTGGTCACTGATT 3'
OPN-canis-R	5' TTGTGGGACTTCTTAGATTTGG 3'
COLI-canis-F	5' GCCAAGAAGAAGACATCCCACCA 3'
COLI-canis-R	5' GCAGATCACGTCATCGCACAACA 3'
OCN-canis-F	5' CAGTGCTGAATCCCGCAAAGGT 3'
OCN-canis-R	5' GCCCAGCCCAGAGTCCAGGTAG 3'

### Haemolytic test

Haemolytic testing was conducted according to the ISO-10993-4:2002 standard [24]. A volume of 4 mL of fresh blood with potassium oxalate (20 g/L) was diluted by 5 mL of physiological saline (0.9% NaCl) from a rabbit under general anaesthesia using 5% chloral hydrate (1 ml/kg) injected intramuscularly. Approximately 5 g EBM, SLM and wrought samples were completely immersed in 10 mL physiological saline in tubes, respectively and kept at 37 ± 1°C in a thermostatic water bath for 30 min. Physiological saline and distilled water with Ti6Al4V samples were employed as negative and positive controls, respectively. Afterwards, 0.2 mL of diluted fresh blood was added into the tubes and kept at 37 ± 1°C in a thermostatic water bath for 60 min. After being centrifuged at 3000 rpm for 5 min, the absorbance of the supernatant solution was measured at 545 nm using a spectrophotometer (Evolution 201/220, USA). The absorbance of the Ti6Al4V samples was recorded Dt. The absorbance of the negative and positive controls was recorded Dnc and Dpc. The haemolytic ratio was calculated using the formula:

Haemolytic ratio = (Dt −Dnc) / (DPC−Dnc) × 100%.

### Dermal irritation test

Dermal irritation testing was performed to assess the potential irritation of EBM and SLM Ti6Al4V samples after topical application. This test was conducted in accordance to the ISO 10993–10:2002 standard [25].

General anaesthesia of three rabbits was performed using 5% chloral hydrate (1 ml/kg) through an intramuscular injection. The fur on both sides of the spine area (approximately 10 cm × 15 cm) was clipped 20 h prior to testing. EBM and SLM Ti6Al4V samples were immersed in physiological saline at 37 ± 1°Cfor 72 h to prepare the leaching liquor. Physiological saline and 10% sodium dodecylsulfate solution (SDS) (Sigma, USA) were applied as negative and positive controls. A test patch was produced using 0.5 mL leaching liquor by dropping it on the surface and moistening with a gauze patch. The test area was divided into four sections: left top, left lower, right top and right lower. To these sections, EBM Ti6Al4V, positive, negative and SLM Ti6Al4V patches were applied individually. The gauze patches were secured with non-irritant semi-occlusive tape (3M, USA). The entire testing area was wrapped with elastic stocking to obviate patch dislocation. After application for 4 h, the gauze patches were removed from the test area, labelled, lightly washed with water to clean any residual test substance. The skin condition was observed and scored for 1 h, 24 h, 48 h and 72 h after gauze patch removal. The skin reaction was scored and the dermal irritation degree was evaluated by calculating the primary dermal irritation index (PII) (Tables 2 and 3). The formula used for this was:

PII = primary dermal irritation score (aggregation score of each points) / total time points.

**Table 2:** Scoring System for Skin Reaction.

Skin Reaction	Skin Reaction
**Erythema and eschar formation**	
No erythema	0
Very slight erythema (barely perceptible)	1
Well defined erythema	2
Moderate erythema	3
Severe erythema (beet-redness) to eschar formation preventing grading of erythema	4
**Oedema formation**	
No oedema	0
Very slight oedema (barely perceptible)	1
Well-defined oedema (edges of area well defined by definite raising)	2
Moderate oedema (raised approximately 1 mm)	3
Severe edema (raised more than 1 mm and extending beyond exposure area)	4
Total possible score for irritation	8

**Table 3:** Irritation Response Categories in Rabbit.

Mean Score	Response Category
0–0.4	Negligible
0.5–1.9	Slightly
2–4.9	Moderate
5–8	Severe

### Delayed-type hypersensitivity test

The classic delayed-type hypersensitivity test and sensitivity methods were used to evaluate the skin sensitivity potential. In this study, a maximization test was used to assess the skin sensitization from the EBM and SLM Ti6Al4V samples and conducted according to the ISO10993-10: 2002 standard [25]. Thirty albino guinea pigs were randomly divided into four groups, ten in the EBM and SLM groups, respectively, and five in the positive and negative groups, respectively.

EBM and SLM Ti6Al4V samples were immersed in physiological saline at 37 ± 1°C for 72 h to produce test extract (1.25 mL/cm2). Physiological saline and α-hydroxy-cinnamon-aldehyde (α-HCA) (Aldrich, USA) solution was applied as negative and positive controls. Due to the undiluted extract used in this study, a pilot study was not necessary. All the animals were placed under general anaesthesia induced by a 2% (w/v) pentobarbital sodium intraperitoneal injection (0.5 mL per animal).

For the intradermal induction phase: Fur between the shoulder (approximately 4 cm × 6 cm) area was clipped using an electric shaver with a chemical depilatory and cleaned with mild water. The prepared skin was divided into three pairs of 0.1 mL intradermal injection sites: A, B and C. The A sites were injected with a stable emulsion of Freund's complete adjuvant (FCA, Sigma, USA) mixed with physiological saline (equal volume ratio). The B sites were injected with undiluted test extract (the control groups were injected with solvent alone). The C sites were injected with mixtures of A and B in an equal volume ratio.

Topical induction phase: Seven days later, the intradermal injection sites were covered with 2 cm × 3.5 cm filter papers. The filter papers were soked from the concentration taken from the site B in each group. The patches were secured with occlusive tapes and wrapped with elastic tapes. After 24 h, the tapes and patches were removed. Challenge phase: Fourteen days after completion of the topical induction phase, the fur on the upper flank of all the animals was clipped and treated with patches soaked from the concentration taken from site C. The patches were secured with occlusive dressing for 24 h. Then, the patches and dressing were removed. The appearance of the skin at the challenge sites in all the animals was observed and scored for 24 and 48 h after patch removing according to the Magnusson and Kligman grading (Table 4). The grades for the test groups were 1 or greater and were less than 1 in the negative control group, indicating a sensitization response. If the grade in the control group was greater than 1, the grade in the test groups exceeded the most severe response in the control group, indicated sensitization.

**Table 4:** Magnusson and Kligman scale.

Patch Test Reaction	Grading Scale
No visible change	0
Discrete or patchy erythema	1
Moderate and confluent erythema	2
Intense erythema and swelling	3

### Statistical analysis

The results were represents as the mean ± standard deviation. The difference between the groups was analysed by one-way ANOVA. The difference between the two groups was analysed by Tukey’s multiple comparison Test. A P value less than 0.05 was considered to be significantly different.

## Results

### Microstructures of Ti6AL4V specimens

**Fig 1:**
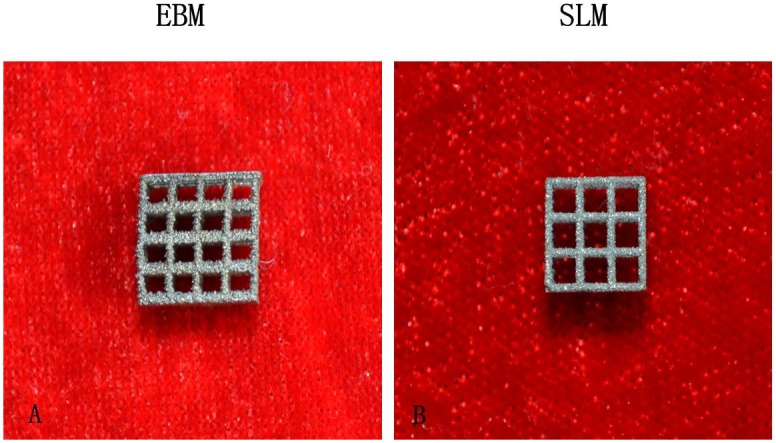
Meshy Ti6AL4V samples fabricated by EBM and SLM. A EBM sample B SLM sample.

**Fig 2:**
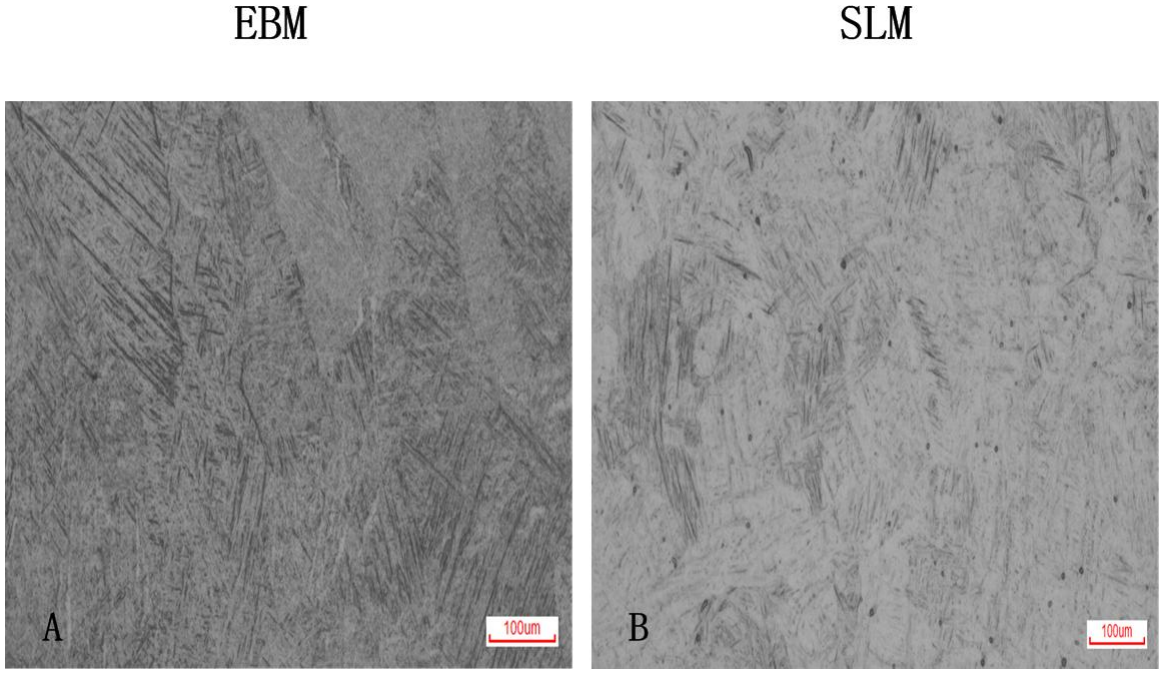
Optical micrographs of EBM and SLM samples. A EBM showed fine α-phase structure. Dark was β phase. B SLM showed dominant α’ martensite.

The meshy structures of Ti6AL4V specimens fabricated by EBM and SLM were showed in Fig 1. Both structures displayed interconnect porosity. The microstructure of EBM specimen was fine α-phase structure with small amount of β phase whereas the microstructure in SLM was dominant α’—martensite plates (Fig 2). The α-phase in EBM was larger than α’—martensite plates in SLM.

### Cell proliferation

**Fig 3:**
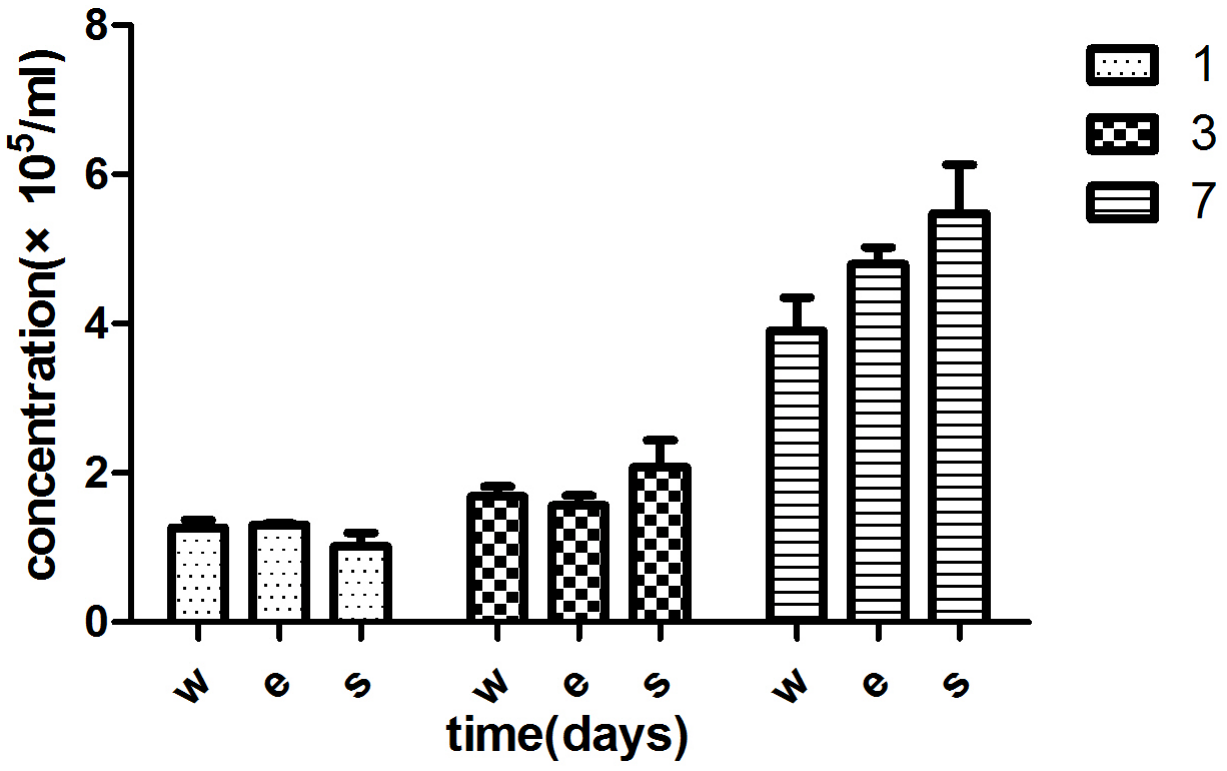
MSC concentrations for each group at 1, 3 and 7 days. MSC concentrations of each group increased with time (p<0.05). There was no significant difference between the three groups (p>0.05).

The proliferation of the MSCs was detected by cell counting after being incubated for 1, 2 and 3 days. Cell numbers on the different Ti6AL4V specimens were counted by count star, and the results were listed in Table 5 and Fig 3. The MSCs of each group increased with time (*p<0.05). There was no significant difference in the three groups at each time point (**p>0.05). However, the cell concentration in SLM groups was higher than EBM groups at 3 and 7 days. The difference was not statistically significant (p>0.05). The EBM and SLM Ti6AL4V specimens did not affect the cell proliferation.

**Table 5:** Cell concentration of each group at different time (days).

Specimen	1	3	7	
w	1.267±0.183	1.687±0.220	3.903±0.770	*
e	1.303±0.038	1.567±0.230	4.797±0.389	*
s	1.02±0.3040	2.073±0.626	5.477±1.136	*
	**	**	**	

*p<0.05,

**p>0.05

### Cell Viability

**Fig 4:**
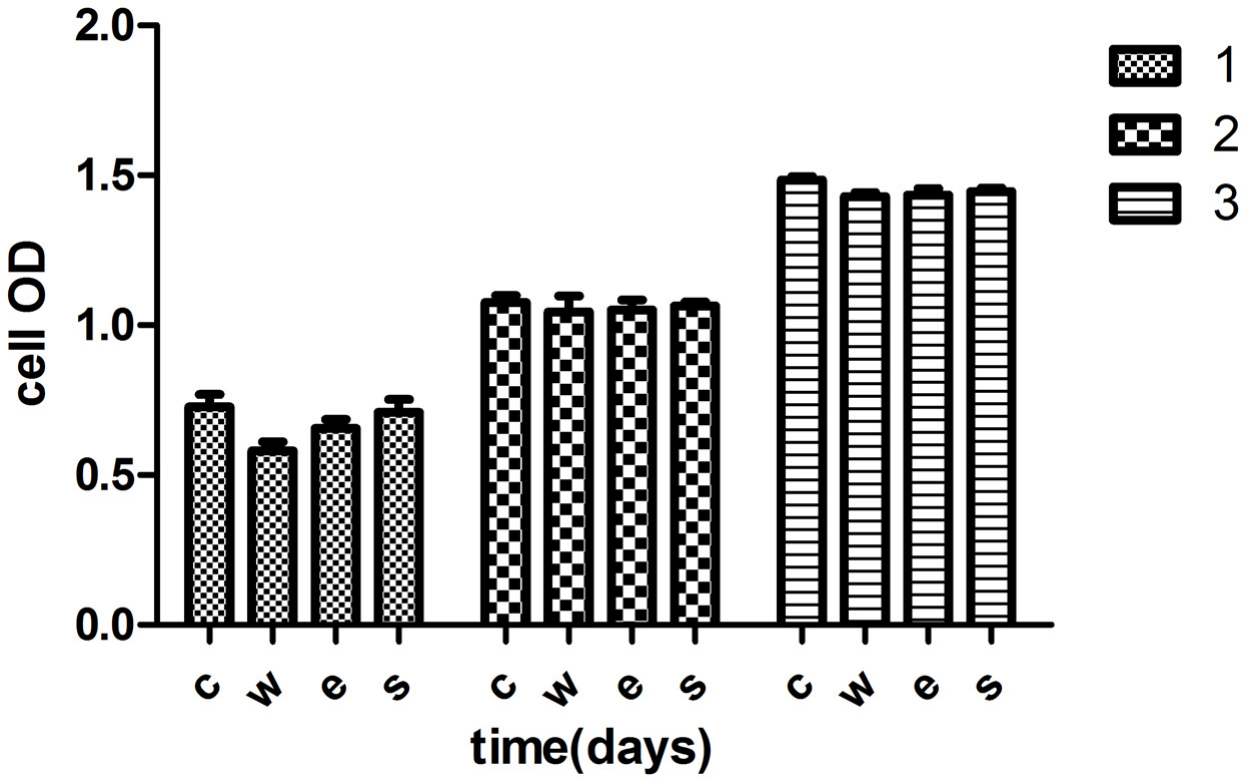
OD in each group at 1, 2 and 3 days. The OD value increased with time (p<0.05). There was no significant difference between the three groups (p>0.05).

The effect of different Ti6AL4V specimens on cell viability was determined by a CCK-8 assay after culturing in leaching liquor for 1, 2 and 3 days (Table 6 and Fig 4). The OD in each group significantly increased with time, reflecting an increase in cell viability (*p<0.05). Cell viability for the normal medium group was higher than for the other three Ti6AL4V groups, as observed from the data. There was no statistically significantly difference (**p>0.05). The OD for the SLM group was slightly higher than that in EBM group. However, there was no significant difference between the EBM and SLM groups (p>0.05). The EBM and SLM Ti6AL4V extract did not affect cell viability.

**Table 6:** Cell OD in each group at different time (days).

Groups	1	2	3	
c	0.728±0.093	1.075±0.055	1.484±0.027	*
w	0.580±0.066	1.044±0.119	1.430±0.028	*
e	0.565±0.038	1.051±0.073	1.435±0.047	*
s	0.565±0.038	1.051±0.073	1.435±0.047	*
	**	**	**	

*p<0.05,

**p>0.05

### Cell attachment and morphology

**Fig 5:**
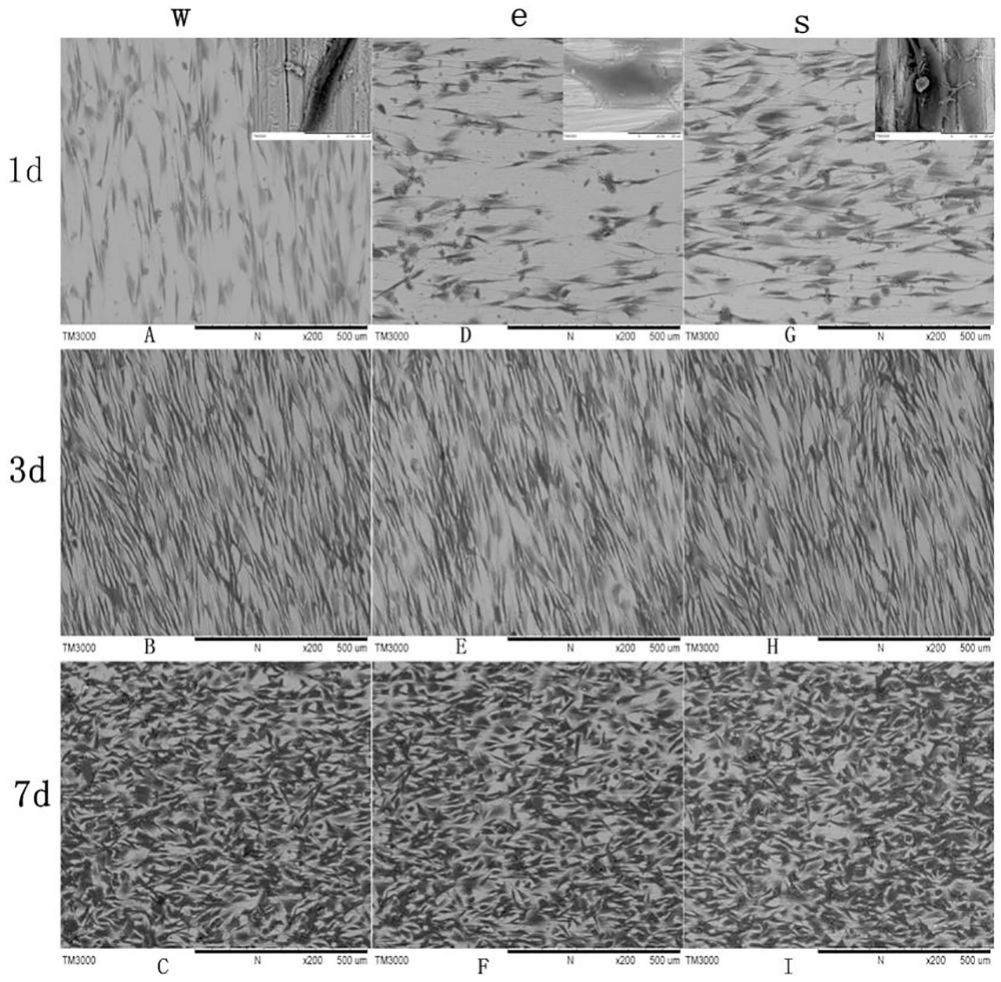
MSCs seeded on the samples for the three groups. The MSCs attached and spread well on the Ti6Al4V samples. The cells density markedly increased with time.

Cell attachment and morphology was investigated by SEM after being cultured on the different Ti6AL4V specimens for 1, 3 and 7 days. The SEM images showed MSC cell exertion with more filopodia, which is a sign of good attachment to these metal samples after 1 day. At 3 days, the cells became slender and began to contact the surrounding cells, which demonstrated that the cells stretched out and grew well (Fig 5). At 7 days, the cells appeared cuboidal and exhibited confluenced flakiness. Cell density was significant increased with time. There was no difference in the morphology of the MSC between the three Ti6Al4V samples. The results demonstrated that Ti6Al4V samples fabricated using EBM and SLM have good biocompatibility, matching that of the wrought group.

### ALP activity

**Fig 6:**
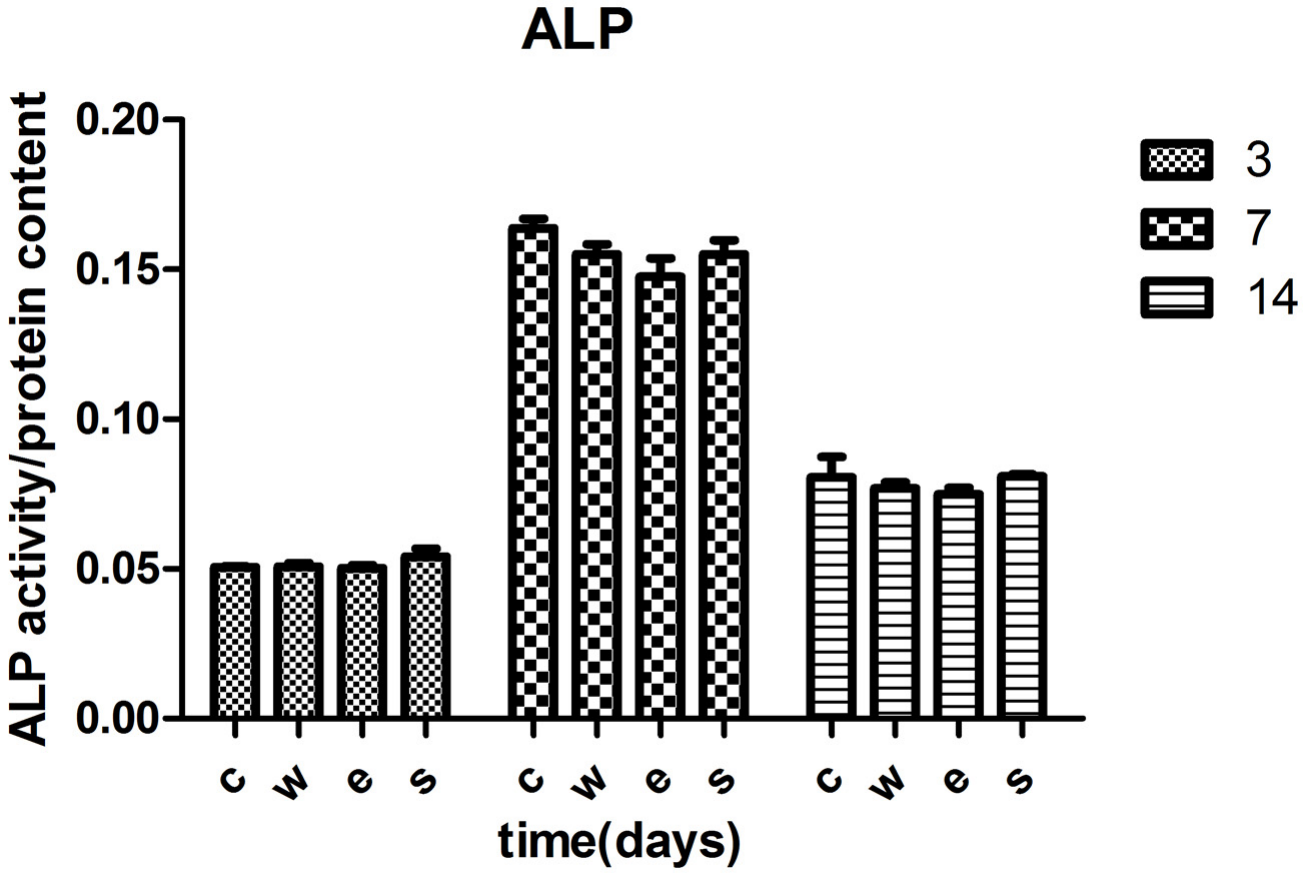
ALP activity of the MSCs at different times. The ALP activity was highest at 7 days under osteogenic induction. The ALP activity decreased after 14 days (p<0.05). There was no statistical difference between the three groups (p>0.05).

After culturing in osteogenic leaching liquor for 3, 7 and 14 days, the ALP activity of the MSC cells was investigated using an ALP quantity kit. The ALP activity was investigated after 3 days under osteogenic induction and was lowest at 3 days, highest at 7 days and began to decrease at 14 days (*p<0.05). There was no difference between the three Ti6Al4V samples (**p>0.05) (Table 7 and Fig 6) However, the ALP activity in the SLM group was slightly higher, which was in contrast to the EBM group at each time point. There was no statistical significance (p>0.05). The SLM and EBM Ti6Al4V samples did not affect the osteogenic ability of the MSC cells.

**Table 7:** ALP activity of MSC cells at different time(days).

Groups	3	7	14	
c	0.0522±0.0032	0.1638±0.0053	0.0805±0.0120	*
w	0.0515±0.0038	0.1550±0.0059	0.0770±0.0035	*
e	0.0510±0.0026	0.1476±0.0107	0.0750±0.0036	*
s	0.0541±0.0045	0.1551±0.0081	0.0809±0.0013	*
	**	**	**	

*p<0.05,

**p>0.05

### Osteogenic gene expression

**Fig 7:**
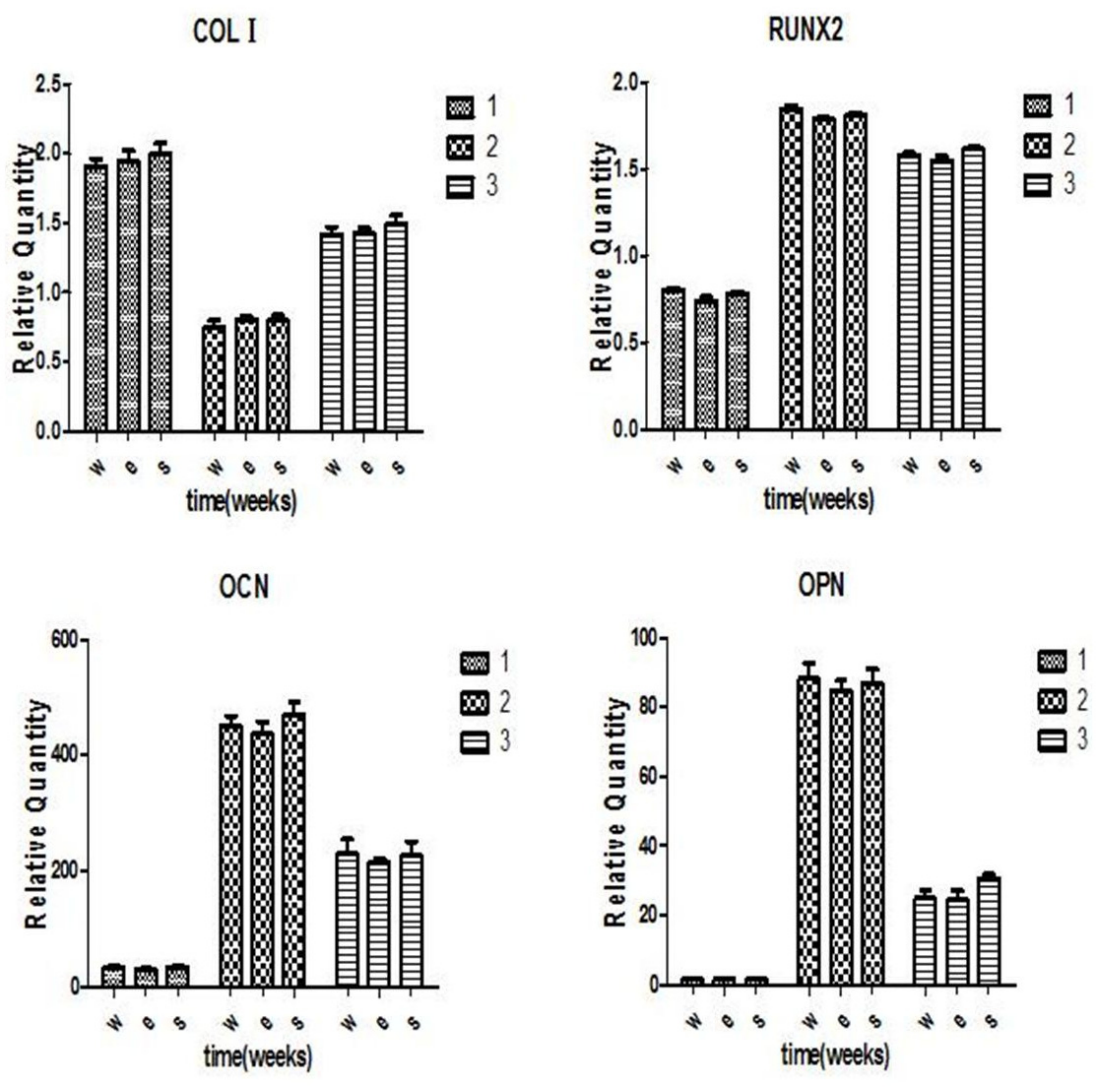
Osteogenic gene expression for the three groups at different times after osteogenic induction. COL I level was highest at 1 week and decreased after 1 week. RUNX2 expression achieved a maximum at 2 weeks. The OCN and OPN expression increased at 1 week and reached a maximum at 2 weeks. There was no significant difference in the four osteogenic gene levels between the three groups. No significant difference was found between the EBM and SLM groups (p>0.05).

To quantify the osteogenic gene expression, we evaluated the effect of the three groups of Ti6Al4V samples on the osteogenic differentiation capacity of the MSCs. The common osteogenic gene was investigated using qPCR. COLIexpression in the three groups exhibited and achieved a maximum early at 1 week after being cultured in the osteogenic medium. The level decreased after 1 week (p<0.05). No difference was observed between the three groups (p>0.05). RUNX2 in the three groups expressed at 1 week after osteogenic induction. RUNX2 expression achieved a maximum at 2 weeks and decreased after 2 weeks in the osteogenic culture (p<0.05). No difference was observed between the three groups (p>0.05). OCN expression was observed at 1 week and reached a maximum at 2 weeks after osteogenic induction. After 2 weeks, its expression decreased. There was no difference between the three groups (p>0.05). OPN expression was similar to OCN. It increased after 1 week and achieved a maximum at 2 weeks under osteogenic induction. No significant difference was observed between the three groups (p>0.05). The osteogenic gene expression in the EBM group was slightly less than for the SLM (Fig 7). However, the difference was not statistically significant.

### Haemolytic Ratio

Haemolytic testing was performed according to the ISO 10993–4 2002 standard. The haemolytic ratio is an important parameter used to evaluate the haemocompatibility of medical materials. A lower haemolytic ratio represents better haemocompatibility. The haemolytic ratio of the wrought, EBM and SLM Ti6Al4V samples was 1.66%, 2.24% and 2.46%, respectively, which was less than the standard value 5%. Both of EBM and SLM Ti6Al4V samples exhibited good blood compatibility. The wrought Ti6Al4V samples had better haemocompatibility compared to the EBM and SLM (p<0.05) specimens. The haemolytic ratio of the SLM was higher than the EBM, which indicated that the EBM Ti6Al4V sample had better blood compatibility than SLM (p< 0.05).

### Dermal irritation test

**Fig 8:**
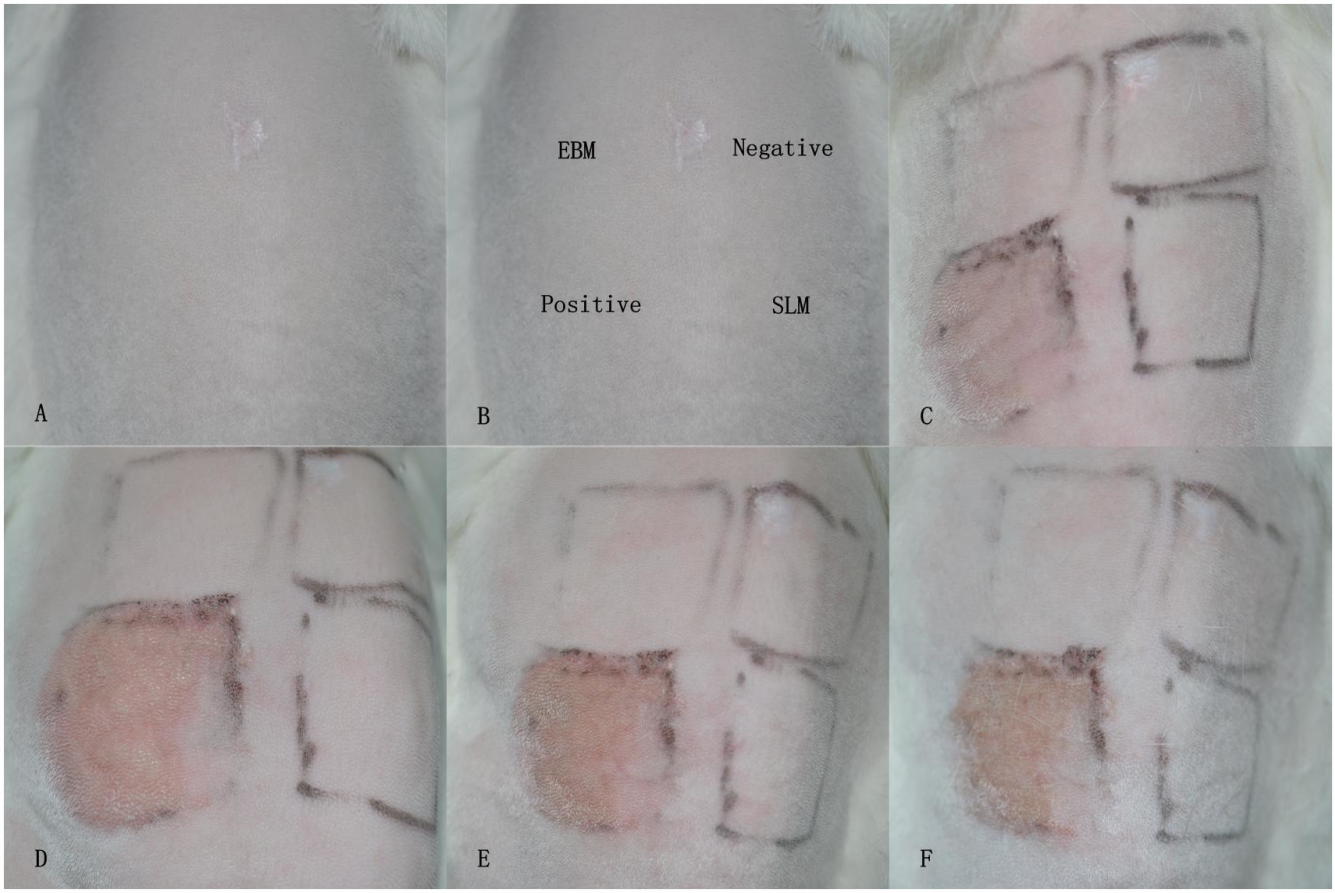
Dermal irritation test. (A) The shaved back and trunk areas. (B) Test area divided into four sections for the application of different test substances. (C) 1 h: perceptible erythema formed in the positive area. By contrast, no erythema was observed in the other areas. (D) 24 h: slight erythema in the positive area. (E) 48 h: moderate erythema and slight oedema in the positive area. (F) 72 h: erythema and oedema alleviated in the positive area. During the test period, no irritation reaction was observed in the EBM and SLM areas, as well as the negative area.

The potential skin irritation was evaluated by dermal irritation testing. The three rabbits were healthy and active without any abnormal reactions through the experimental period. No erythema, eschar and oedema formed in the EBM and SLM groups as well as the negative control group. PII for the three groups was scored as 0. In contrast, erythema and oedema did form in the positive group and, PII was scored as 3. The results in this test indicated that the EBM and SLM Ti6Al4V samples did not cause dermal irritation (Fig 8).

### Delayed-type hypersensitivity test

**Fig 9:**
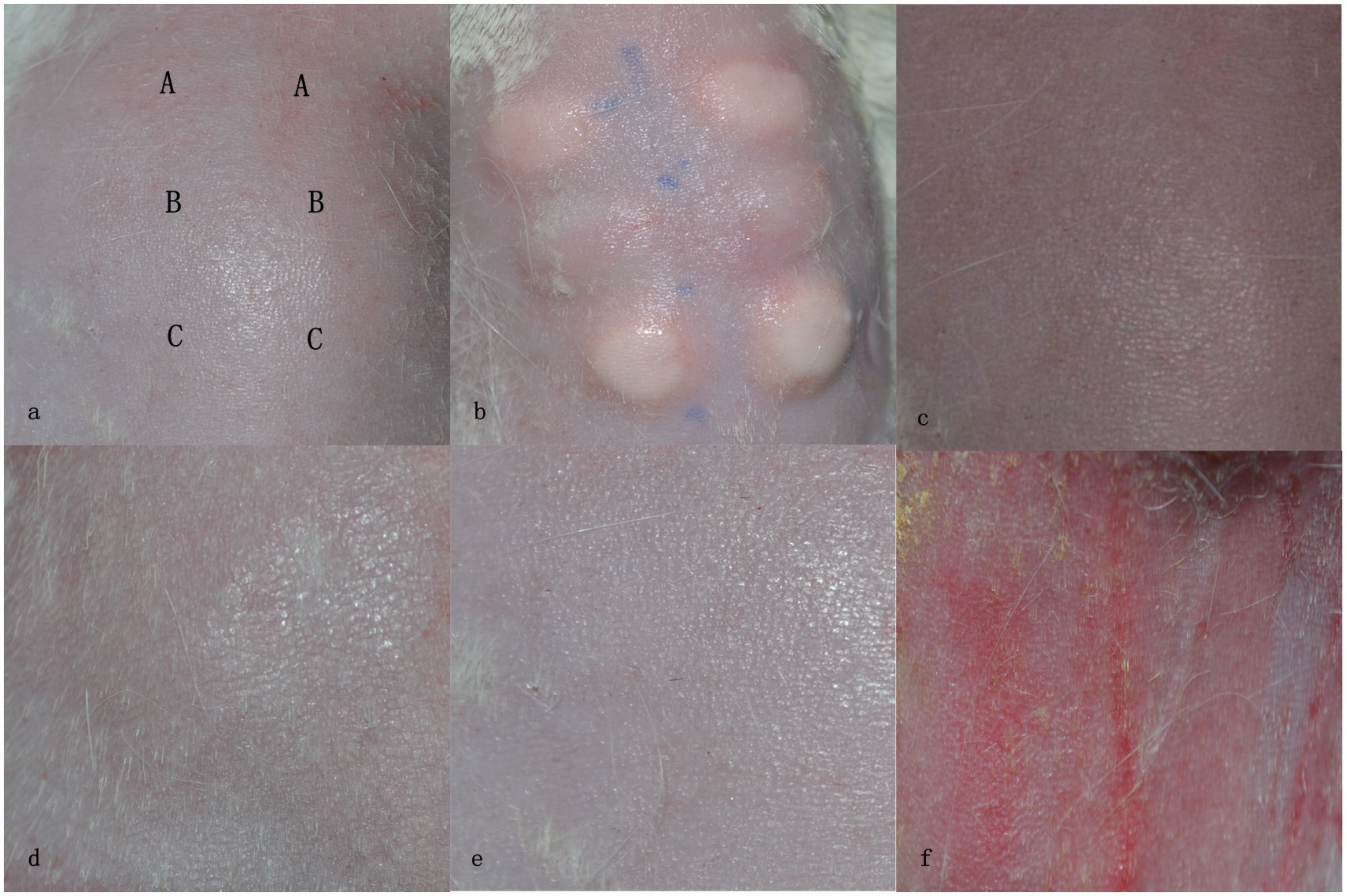
Delayed-type hypersensitivity test. (a) and (b) Intradermal injection sites. (c), (d) and (e) Negative, EBM and SLM groups, respectively. No erythema or other irritation appearance formed in the challenge sites for the three groups. (f) Positive group: discrete or patchy erythema formed in the challenge sites.

Two guinea pigs died for uncorrelated reasons to treatment during the experimental period and another two animals were supplemented. All of the sites treated with α-HCA in the positive control group formed erythema. In contrast, the test sites for the EBM and SLM as well as negative group exhibited no erythema or any other irritation response (Fig 9). The grade of the positive group was 1.1, more than 1. The grades of EBM and SLM groups were 0 (Table 8). Based on these results, the EBM and SLM Ti6Al4V samples did not provoke skin sensitization in guinea pigs.

**Table 8:** Response to sensitization in guinea pigs.

Samples	Magnusson and Kligman scale (average)	Results
Negative	0	negative
Positive	1.1	positive
EBM	0	negative
SLM	0	negative

## Discussion

Metallic materials, especially titanium and its alloys, are extensively used in bone substitution, dentistry, orthotics, cranio-maxillofacial surgery and replacement arthroplasty due to their light weight, excellent mechanic properties, corrosion resistance and good biocompatibility [26–31]. Compared to traditional fabrication methods of metallic materials, EBM and SLM are two advanced direct metal fabrication techniques, which are optimal choices for complex structured implants. In addition, porous metallic scaffolds fabricated by EBM and SLM have completely interconnected and possessed the modified pore size, pore shape and proper mechanical strength suitable for bone tissue engineering [32–33].

Studies showed different fabrication process between EBM and SLM including power efficiency, powder size and fabrication parameters result in different microstructure, mechanical and chemical properties which are closed to biocompatibility of titanium alloy [10–15]. For clinical applications, biocompatibility is the fundamental essential for metallic materials. Therefore, the biocompatibility of EBM and SLM fabricated products needs to be assessed. Some studies had investigated the biocompatibility of EBM or SLM titanium alloy products [9,21,34]. However, the comparison of biocompatibility between EBM and SLM products was little know. In this study, we assessed and compared the biocompatibility of EBM and SLM Ti6Al4V products.

We examined the microstructures of the EBM and SLM Ti6AL4V specimens. EBM specimem has fine α-phase structure with small β phase and SLM was dominant α’—martensite plates. The α-phase in EBM was larger than α’ martensite plates of SLM (Fig 1). The results were coincidence with other studies[10–15]. Koike et al described EBM Ti6Al4V consist of prominent acicular α-plates, nevertheless, SLM Ti6Al4Vconsist of mixture of α-phase and α’ martensite [13].

MSCs derived from bone marrow are considered better stem cells for bone tissue engineering [35]. As bone substitutes or bone tissue engineering scaffolds, the effect that the EBM and SLM Ti6Al4V had on the proliferation and osteogenic differentiation ability of the MSCs was investigated. The interaction between the Ti6Al4V implantation and the MSCs includes cells attachment, proliferation and osteogenic differentiation. We investigated the cells attachment and proliferation using direct and indirect methods.

Titanium alloy surface plays an important role in the attachment, proliferation and differentiation of cells. Studies noted that the surface roughness of titanium alloy samples by EBM and SLM was different [14,36,37]. In order to eliminate the effect of surface roughness, the polished surface of Ti6Al4V samples were used. The data of cells counting and CCK-8 addressed that MSCs grow well and proliferated with time extended on three Ti6Al4V samples indicated Ti6Al4V samples with EBM and SLM have no toxic effect on MSCs.

Fig 4 showed MSCs stretched out completely and arranged parallelly on Ti6Al4V samples. MSCs exhibited more filopodia 24 h after seeding on Ti6Al4V samples. With time extended, the density of MSCs on Ti6Al4V samples was significantly increased and cells fused into layer, as observed from the SEM image. This result demonstrated that all of the Ti6Al4V samples have good adhesiveness with regards to the MSCs. In this perspective, the EBM and SLM Ti6Al4V samples have good cytocompatibility and are the same as the wrought samples. Our results are in agreement with the studies reported by Peppo [21], Haslauer [34] and Warnke et al [9]. Peppo and Haslauer et al investigated the in vitro biocompatibility of porous EBM-produced Ti6Al4V with human embryonic stem cell-derived mesodermal or human adipose-derived adult stem cells, respectively. Both studies suggested that EBM Ti6Al4V support cell attachment and growth. Warnke et al cultured human osteoblasts on SLM-produced Ti6Al4V mesh scaffolds to evaluate biocompatibility. They concluded that SLM-produced Ti6Al4V scaffolds allow cell growth and exhibit good biocompatibility [9].

Osteogenic differentiation of MSCs is the base for bone regeneration. However, the corrosion of Ti6Al4V implants in the body is inevitable due to long-term contact with bodily liquids [38]. Experiments have reported that elevated titanium ion concentrations can induce osteoclast differentiation, which may relate to aseptic loosening of titanium orthopaedic implants [16]. As bone tissue engineering scaffolds, it needs to be determined if Ti6Al4V samples produced by EBM and SLM affect the osteogenic ability of MSCs. Bone regeneration processes include MSCs differentiated into osteoblasts, and the secretion and mineralization of the bone extracellular matrix. In this study, we investigated the influence of Ti6Al4V samples on the osteogenic differentiation of MSCs by measuring the ALP activity and common osteogenic gene expression. ALP is an early signs of osteogenic differentiation [39]. In our study, the ALP expressed as early as 3 days after osteogenic culturing. ALP activity was achieved a maximum at 7 days and decreased after 14 days. This tendency in the three groups was concurrent, which indicated the osteogenic differentiation of MSCs was not inhibited by Ti6Al4V samples manufactured using SLM and EBM. COL Iis also regarded as a typical early marker of osteogenic differentiation [40]. The data from this study showed that COLIhighly expressed at the early stage of the MSCs osteogenic differentiation. No significant difference was found between three groups. RUNX2 is the major transcription factor of osteogenic differentiation for the MSCs, which regulates the osteogenic related genes including, ALP, COL 1, OCN, OPN and BSP [41–44]. The RUNX2 expression level was increased at 1 week, reached a high at 2 weeks and decreased at 3 weeks, which indicated that RUNX2 is an early osteogenic gene. This also demonstrated the osteogenic differentiation in the MSCs was normal in our study.

The OCN and OPN are representative osteogenic markers and are expressed during the mineralization stage of MSC osteogenic differentiation [42,45–46]. In our study, the OCN and OPN expression level was low at 1 week, increased at 2 weeks and decreased at 3 weeks after osteogenic induction. These results were different from the data from Peppo et al [21]. The level of OCN and OPN in their study was significantly expressed at 1 week after osteogenic induction. The reason might be due to the porous structure of Ti6Al4V samples or a different cell source. Osteogenic ability comparison results suggest that the EBM and SLM Ti6Al4V samples do not present any adverse effect on the osteogenic differentiation of the MSCs. Nevertheless, the osteogenic ability in SLM material was higher than in the EBM material. However, there are no significant differences between the two groups.

Haemolysis is a significant screening test used to evaluate the blood compatibility of medical materials. The haemolytic reaction triggered by toxic materials is more sensitive than cellular reactions [24]. A low haemolytic ratio represents better haemocompatibility. In our study, the haemolytic ratios for the EBM and SLM Ti6Al4V samples were 2.24% and 2.46%, respectively. Although the haemolytic ratio in SLM Ti6Al4V was higher than in EBM, both were lower than the standard value of 5%, which demonstrated both EBM and SLM Ti6Al4V have good blood compatibility. Haemolysis is related to the shape, surface roughness and soluble components of biomaterials. Identical shape and surface roughness of Ti6Al4V samples were used in this study. So, the difference of haemolysis in EBM and SLM was due to different corrossion resistence. Nevertheless, better corrosion of SLM compared to EBM was reported. The reason for this result was unknown and need further studied.

Long-term contact with biological fluids under mechanical action and metallic implant corrosion results in the release of metal ions and wear particles, which may trigger an inflammatory reaction and immunological rejection account for the failure of metallic implants [47–50]. Although titanium and its alloys possess good corrosion resistance, reports of allergic reactions, such as dermatitis, pain, swelling or implant loosening provoked by titanium and its alloys suggest that sensitivity studies need to continue [51–53]. In our study, the results of the dermal irritation test indicated that the EBM and SLM Ti6Al4V samples were non-irritant materials. During the delayed-type hypersensitivity test, no skin reaction, such as erythema or oedema was observed at the test sites for the EBM and SLM Ti6Al4V, revealing that the sensitization results were negative. Both the EBM and SLM Ti6Al4V products did not exhibit skin sensitizing potential. Although the study reported that SLM Ti6Al4V products have a better corrosion resistance than EBM Ti6Al4V products, there was no difference observed in the dermal irritation and skin sensitization tests.

## Conclusions

In summary, the results of the cytocompatibility, blood biocompatibility, skin irritation and skin sensitivity tests indicated that the Ti6Al4V samples fabricated by EBM and SLM have good biocompatibility both in vitro and in vivo. Although the data associated with cell viability, osteogenic ability and the haemolytic ratio was higher for the SLM group, there was no significant difference between the results for the two groups. The variance may be related to different microstructure, physical and chemical properties of the Ti6Al4V fabricated by EBM and SLM. Relevant examinations of these factors are addressed in further study. Additionally, more in vivo and long-term studies are needed to evaluate and compare the biocompatibility of Ti6Al4V implants fabricated using EBM and SLM. The superior fabrication method for metallic materials should be determined to provide an actual requirement for biomedical applications.
